# Aberrant resting-state functional connectivity in incarcerated women with elevated psychopathic traits

**DOI:** 10.3389/fnimg.2022.971201

**Published:** 2022-10-04

**Authors:** Corey H. Allen, J. Michael Maurer, Bethany G. Edwards, Aparna R. Gullapalli, Carla L. Harenski, Keith A. Harenski, Vince D. Calhoun, Kent A. Kiehl

**Affiliations:** ^1^The Mind Research Network, Albuquerque, NM, United States; ^2^Department of Psychology, University of New Mexico, Albuquerque, NM, United States; ^3^Department of Electrical and Computer Engineering, Georgia Institute of Technology, Atlanta, GA, United States; ^4^Tri-Institutional Center for Translational Research in Neuroimaging and Data Science (TReNDS), Georgia Institute of Technology, Georgia State University, Emory University, Atlanta, GA, United States; ^5^Department of Computer Science, Georgia State University, Atlanta, GA, United States

**Keywords:** psychopathy, functional connectivity, intra-network connectivity, spectra, antisocial

## Abstract

Previous work in incarcerated men suggests that individuals scoring high on psychopathy exhibit aberrant resting-state paralimbic functional network connectivity (FNC). However, it is unclear whether similar results extend to women scoring high on psychopathy. This study examined whether psychopathic traits [assessed *via* the Hare Psychopathy Checklist – Revised (PCL-R)] were associated with aberrant inter-network connectivity, intra-network connectivity (i.e., functional coherence within a network), and amplitude of fluctuations across limbic and surrounding paralimbic regions among incarcerated women (*n* = 297). Resting-state networks were identified by applying group Independent Component Analysis to resting-state fMRI scans. We tested the association of psychopathic traits (PCL-R Factor 1 measuring interpersonal/affective psychopathic traits and PCL-R Factor 2 assessing lifestyle/antisocial psychopathic traits) to the three FNC measures. PCL-R Factor 1 scores were associated with increased low-frequency fluctuations in executive control and attentional networks, decreased high-frequency fluctuations in executive control and visual networks, and decreased intra-network FNC in default mode network. PCL-R Factor 2 scores were associated with decreased high-frequency fluctuations and default mode networks, and both increased and decreased intra-network functional connectivity in visual networks. Similar to previous analyses in incarcerated men, our results suggest that psychopathic traits among incarcerated women are associated with aberrant intra-network amplitude fluctuations and connectivity across multiple networks including limbic and surrounding paralimbic regions.

## Introduction

Individuals scoring high on psychopathy are characterized by a constellation of traits including impulsivity, poor decision making, callousness, and a lack of empathy (Hare, 2003). An individual with psychopathy is 20–25 times more likely to be arrested than a non-psychopathic individual, and once released, is four to eight times as likely to recidivate violently 1-year post-release (Hemphill et al., 1998; Kiehl and Hoffman, 2011). Relatedly, the social costs due to elevated criminal activity that can be attributed to individuals with psychopathy are estimated to be nearly $460 billion per year (Anderson, 1999; Kiehl and Hoffman, 2011). As such, great efforts have been made to understand the sociological, psychological, and neurobiological origins of the psychopathic phenotype.

Most theories suggest that individuals scoring high on psychopathy exhibit deficits in limbic (e.g., amygdala, cingulate gyrus, and parahippocampal gyrus) and surrounding paralimbic brain regions (e.g., orbitofrontal cortex, insula, and temporal pole) (Kiehl, 2006; Anderson and Kiehl, 2012; though see Blair, 2006). Resting-state functional analyses suggest broadly distributed psychopathy-related aberrations in inter-network functional network connectivity (FNC). These aberrances span across multiple networks but primarily occur in networks associated with executive control, decision making, salience detection, and motor control (Tang et al., 2013; Contreras-Rodríguez et al., 2015; Del Casale et al., 2015; Philippi et al., 2015; Leutgeb et al., 2016; Korponay et al., 2017; Espinoza et al., 2018; Dotterer et al., 2020).

Psychometric analyses generally support dividing psychopathic traits, assessed *via* the Hare Psychopathy Checklist – Revised (PCL-R; Hare, 2003), into two clusters or factors (Harpur et al., 1989; Hare and Neumann, 2010). Factor 1 contains items related to interpersonal and affective traits, while Factor 2 assesses impulsive, life-course developmental, and antisocial traits. Several studies have found interpersonal and affective traits to be associated with localized disruption between the DMN and central executive network (CEN) (Espinoza et al., 2018; Dotterer et al., 2020). Lifestyle/behavioral and antisocial/developmental psychopathic traits, on the other hand, are associated with resting-state correlates ranging from subcortical structures to sensorimotor networks (SEN), DMNs, and visual networks (VIS) (Korponay et al., 2017). Overall, these findings suggest that specific psychopathic traits may associate differentially with resting-state measures.

The bulk of these previous studies have been conducted on entirely male samples, leaving open the question of sex-specific differences in the neurobiological correlates of psychopathy (Verona and Vitale, 2018). Women scoring high on psychopathy are characterized by similar neurobiological deficits as men scoring high on psychopathy (Carré et al., 2013; Cope et al., 2014; Harenski et al., 2014a; Crooks et al., 2019; Maurer et al., 2022), but unique gender differences have also been observed. For example, while men scoring high on psychopathy are characterized by response perseveration deficits, women scoring high on psychopathy are not (Vitale and Newman, 2001b). As such, women scoring high on psychopathy may be characterized by unique FNC patterns compared to men scoring high on psychopathy. Despite advances in our understanding of the relationship between psychopathy and inter-network connectivity of RSNs, research including analyses of amplitude of fluctuations (AFs)—that is, the spectral power of RSN activational profiles—and intra-network connectivity in their relationship to antisocial traits are scant. Furthermore, these studies are largely group comparison based rather dimensionality based (Liu et al., 2014; Xu et al., 2014; Cao et al., 2018). Intra-network high-frequency AFs are believed to contribute to higher-order cognitive processes, and thus, may also differ dimensionally with psychopathic traits (Baria et al., 2011; Craig et al., 2018). These limitations of scope and study obscure functional aberrances associated with psychopathy that may occur on a local RSN specific level rather than an inter-RSN level, as well as potential dimensional correlates associated with psychopathic traits of interest that may be otherwise lost *via* traditional group comparisons.

Here we examine resting-state measures and their relationships to psychopathic traits (assessed *via* the PCL-R) (Hare, 2003) in a large sample of incarcerated women (*n* = 297). Functional connectivity was assessed using three different measures [static functional network connectivity (sFNC: inter-network connectivity), AFs, and intra-network connectivity], to comprehensively evaluate the functional characteristics of RSNs and their associations with psychopathic traits in women. We hypothesized that the majority of aberrant functional connectivity measures related to psychopathy would occur in limbic and paralimbic regions of the brain (Kiehl, 2006).

## Methods

### Participants

Participants included 308 adult female offenders recruited from a medium- and maximum-security correctional facility who previously participated as part of NIH-funded research and treatment studies (R01 DA020870, R01 DA026964, and R01 MH085010). While all offenders within the correctional facility were offered the opportunity to participate in the current study, the final sample included participants who completed the relevant clinical assessments and resting-state functional MRI scans and met further inclusion criteria. Inclusion criteria included fluency in English at or above a fourth-grade reading level; estimated IQ over 70 (*n* = 4 excluded); and no presence of psychotic disorder (schizoaffective disorder, *n* = 1; delusional disorder, *n* = 1, excluded). An additional *n* = 5 participants were excluded for MRI-related reasons, excessive head motion (i.e., mean framewise displacement values > three standard deviations above the mean or comprising more than 10% of their total volume, *n* = 3; Power et al., 2014), large susceptibility artifacts (*n* = 1), or an incomplete resting-state scan (*n* = 1). In total, 11 (3.57%) participants were excluded from the study, leaving a final sample of 297 adult female offenders.

Participants were between the ages of 21 and 57 (average age = 34.6 years, SD = 7.5 years) at the time of their scan and ~10% were left-handed. Based on NIH racial and ethnic classification, 78.5% of the sample self-identified as White, 9.1% as Black/African American, 9.1% as American Indian or Alaskan Native, 3.4% as mixed/other, and 56.2% as Hispanic. Participants' demographics and PCL-R scores are shown in Table 1. Participants provided written informed consent in protocols approved by the institutional review board of the University of New Mexico and by the Independent Review (E&I) Services for the Mind Research Network and were paid at a rate commensurate with institution compensation for work assignments at their facility.

**Table 1:** Participants demographic, PCL-R scores, and disorder rates.

	**Mean**	***SD***	**Min**.	**Max**.	**Overall sample (%)**
Age (years)	34.6	7.5	21	57	
IQ	94.7	9.9	72	123	
PCL-R total scores	18.8	6.2	2.2	34.0	
Factor 1 scores	4.6	2.7	0	13.0	
Factor 2 scores	12.2	3.8	0	20.0	
Any mood disorder					36.4
Any anxiety disorder					12.5
PTSD					7.1
Any substance use disorder					96.0

Four participants were missing mood disorder data, 10 were missing anxiety disorder data, eight were missing PTSD data, and two were missing substance use disorder data.

### Psychopathy scores

Psychopathic traits were assessed using the PCL-R (Hare, 2003), which has been validated for use among women (Vitale and Newman, 2001a; Vitale et al., 2002). The PCL-R consists of 20 items which are scored on a three-point scale, 0 (does not apply), 1 (applies somewhat), and 2 (definitely applies). It is based on participants' clinical interview and extensive file review conducted by trained research staff. The resulting PCL-R total scores range from 0 to 40. Factor analyses of the 20 PCL-R items have consistently revealed two factors: Factor 1 scores correspond to affective/interpersonal characteristics (e.g., manipulativeness, deficient empathy, and a lack of remorse), whereas Factor 2 scores, correspond to impulsive and irresponsible behavior and early and persistent antisocial behavior (Harpur et al., 1989; Hare and Neumann, 2010). While the two-factor model of psychopathy was originally developed and validated in men (Harpur et al., 1989; Hare and Neumann, 2010), research suggests similar validity in women (Kennealy et al., 2007), including participants included in the current sample (Eisenbarth et al., 2018).

### Additional psychosocial data

#### Mood, anxiety, post-traumatic stress, and substance use disorders

Participants were assessed for past or current presence of a mood disorder, including major depressive disorder, dysthymic disorder or persistent depressive disorder, depressive disorder not otherwise specified (NOS), bipolar disorder, mood disorder due to a general medical condition (GMC), and substance-induced mood disorder using the Structured Clinical Interview for DSM-IV-TR Axis I Disorders (SCID-I/P; First et al., 2002) or Structured Clinical Interview for DSM-5–Research Version (SCID-5-RV; First et al., 2015)^1^. Likewise, participants were assessed for lifetime presence of an anxiety disorder (i.e., panic disorder, agoraphobia, social phobia or social anxiety disorder, specific phobia, generalized anxiety disorder, anxiety disorder NOS or otherwise specified, anxiety disorder due to a GMC, and substance-induced anxiety disorder). Finally, participants were also assessed for lifetime posttraumatic stress disorder (PTSD). PTSD assessment procedures differed across SCID versions, and when the SCID-I/P was used, participants completed an initial screening form to determine whether the PTSD interview module would be administered in full. When the SCID-5-RV version was used the PTSD interview module was administered to all participants. Presence of any mood, anxiety, or traumatic disorder was coded dichotomously, and participants meeting past or current diagnostic criteria for any one of these disorders were coded as having the respective disorder (see Table 1).

Alcohol and/or substance-related diagnoses were assigned based on diagnostic criteria for the SCID version administered (either the SCID-I/P or the SCID-5-RV). Participants administered the SCID-I/P were assessed for lifetime alcohol and drug abuse or dependence. A lifetime diagnosis of abuse was defined as scoring at threshold on at least one of four abuse criteria for alcohol and/or seven drug categories (i.e., sedatives-hypnotics-anxiolytics, cannabis, stimulants, opioids, cocaine, hallucinogens/PCP, and other). A lifetime diagnosis of dependence was defined as scoring at threshold in at least three of seven dependence criteria. Alternatively, for participants administered the SCID-5-RV, a lifetime alcohol use disorder was obtained if at least two of 11 alcohol criteria were scored at threshold, and lifetime substance use disorder(s) were obtained if at least two of 11 criteria were scored at threshold for eight categories (i.e., sedative-hypnotics-anxiolytics, cannabis, stimulants, opioids, inhalants, PCP, hallucinogens, and other/unknown; see Table 1).

### Imaging parameters

Resting-state functional magnetic resonance images were collected on the grounds of the correctional facility where participants were housed using the Mind Research Network's mobile Siemens 1.5 T Avanto with advanced SQ gradients (max slew rate 200 T/m/s, 346 T/m/s vector summation, rise time 200 us) equipped with a 12-element head coil. The EPI gradient echo pulse sequence (TR = 2,000 ms, TE = 39 ms, flip angle = 75, FOV = 24 x 24 cm, 64 x 64 matrix, 3.75 x 3.75 mm in-plane resolution, 4 mm slice thickness, 1 mm gap, 27 slices) effectively covered the entire brain (150 mm) in 2.0 s. Head motion was minimized using padding and restraint. The participants were asked to lay still, look at the fixation cross and keep eyes open during the 5-min rsfMRI scanning. Compliance with instructions was monitored by eye tracking.

### EPI preprocessing

Data were preprocessed using statistical parametric mapping (SPM12) (Friston et al., 1994) (http://www.fil.ion.ucl.ac.uk/spm) including image reorientation, realignment [motion estimation using INRialign (Freire and Mangin, 2001)], and spatial normalization to the Montreal Neurological Institute standard space at a resolution of a 3 x 3 x 3 mm^3^. A full width half maximum Gaussian kernel of 6 mm was then used for spatial smoothing. Framewise displacement (FWD) was used to assess motion quality control. For FWD, the translation and rotation parameters were computed as the mean of the sums of the absolute translation and rotation frame displacements, yielding a single FWD value for each participant.

### Independent component analysis

Per Espinoza et al. (2018), we applied gICA on the preprocessed rsfMRI data using the GIFT toolbox (http://trendscenter.org/software/gift) (Calhoun et al., 2001). The rsfMRI data was compressed using two stages of principal component analysis (PCA) (Rachakonda et al., 2016). For the first data reduction step, we retained 100 principal components (PCs), and 75 independent components (ICs) for group data reduction, consistent with previously published studies (Kiviniemi et al., 2009; Smith et al., 2009; Ystad et al., 2010; Allen et al., 2011a; Elseoud et al., 2011; Erhardt et al., 2011). High-model order ICA (i.e., 75 components) results in more refined components corresponding to known anatomical and functional segmentations compared to low-model order ICA (i.e., 25 or 50 components) (Allen et al., 2011a; Hu et al., 2020). Individual specific spatial maps and their time-courses were obtained using gICA. Out of the 75 ICs that were estimated, 48 components were identified as components of RSNs by evaluating whether peak activation occurred in gray matter and whether the peak AFs occurred in the low-frequency power portion of the spectra of components (Meda et al., 2008; Robinson et al., 2009; Allen et al., 2011a). The reliability and stability of these extracted networks were evaluated *via* ICASSO (Himberg and Hyvärinen, 2003), a process that iteratively re-runs component estimations with differently bootstrapped datasets. This analysis suggested high stability across the 48 components (*mean stability index* = 0.89), well above the threshold of 0.70 established in the literature (Ma et al., 2011). The other 27 components were excluded, as they appeared to be related to motion artifacts, spatial maps including white matter, the ventricular system, or cerebrospinal fluid, or having irregular time-course spectra power (Allen et al., 2011a,b). Within GIFT, the time-courses of the RSNs underwent despiking and bandpass by filtering with [0.01–0.15] Hz cutoffs.

### Functional connectivity measures

In order to assess various types of resting-state functional connectivity measures, we calculated the sFNC between the selected 48 RSNs as pairwise correlations between the RSNs time-courses for each individual (inter-network connectivity), pairwise correlations between individual voxels within the RNSs to the overall RSN's time-course (intra-network connectivity), and the AFs within each RSN.

### Statistical analyses

We performed regression analysis to identify associations between individual sFNC values (inter-network connectivity), spatial maps (intra-network connectivity), and AFs with psychopathy measures: PCL-R Factor 1 and Factor 2 as continuous variables (see Supplementary materials for analyses of PCL-R total). The analyses were corrected for “nuisance” covariates (age, IQ^2^, FWD^3^). The significance of the univariate psychopathy results for each factor was determined using a false discovery rate (FDR) (Genovese et al., 2002) threshold at *p* < 0.05.

## Results

### Psychopathic traits

The PCL-R total scores for this sample ranged from 2.2 to 34.0 (mean = 18.8, SD = 6.2, Cronbach's α = 0.79; see Table 1). PCL-R Factor 1 scores ranged from 0.0 to 13.0 (mean = 4.6, SD = 2.7; see Table 1), and PCL-R Factor 2 scores ranged from 0.0 to 20.0 (mean = 12.2, SD = 3.8; see Table 1).

### Group independent component analysis and group level inter-network connectivity

**Figure 1:**
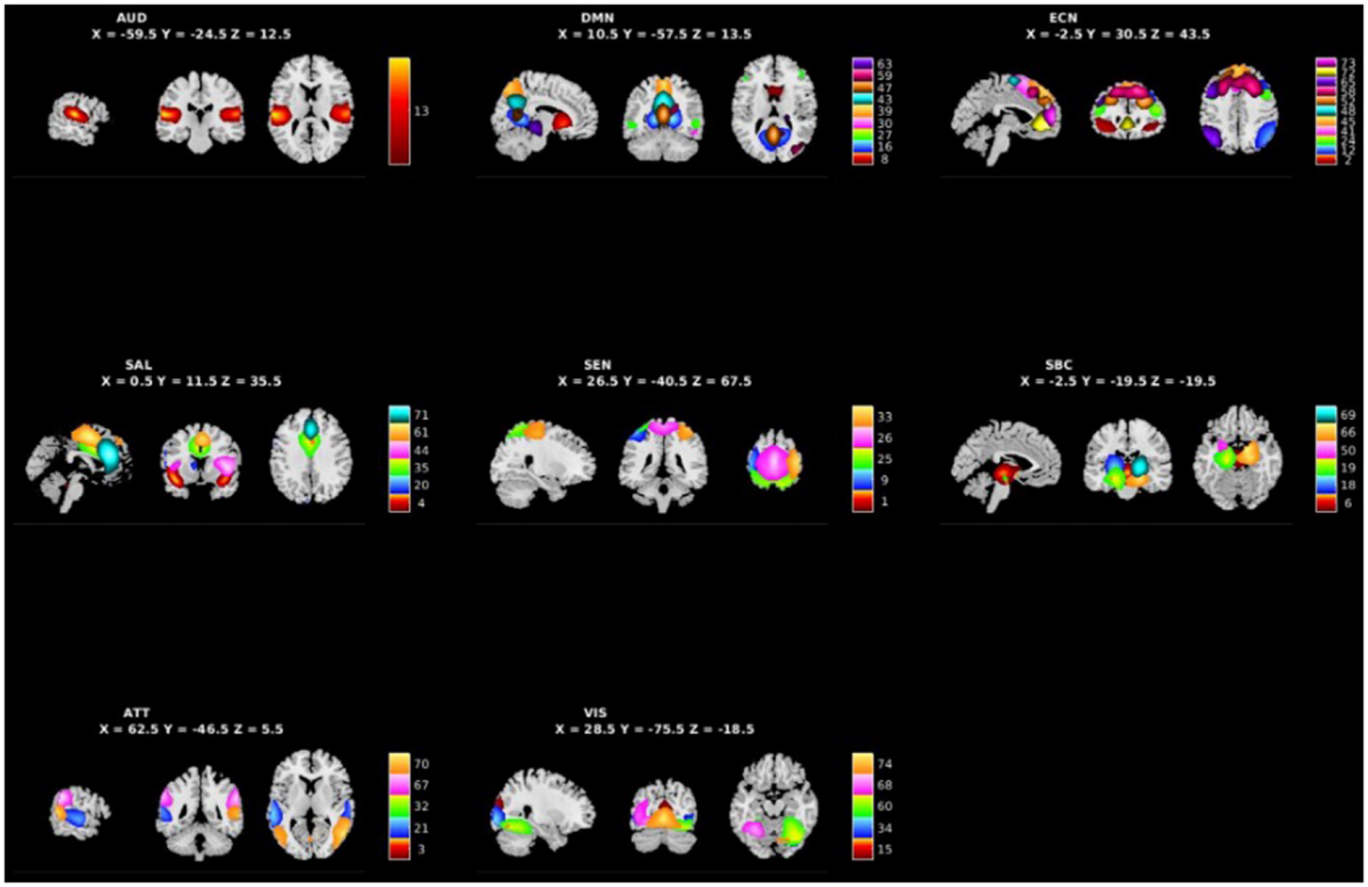
Spatial maps of the 48 independent components identified as RSNs categorized by domain [auditory (AUD), default mode network (DMN), executive control (ECN), salience (SAL), sensorimotor (SEN), subcortical (SBC), attentional (ATT), and visual (VIS)] and component number.

**Figure 2:**
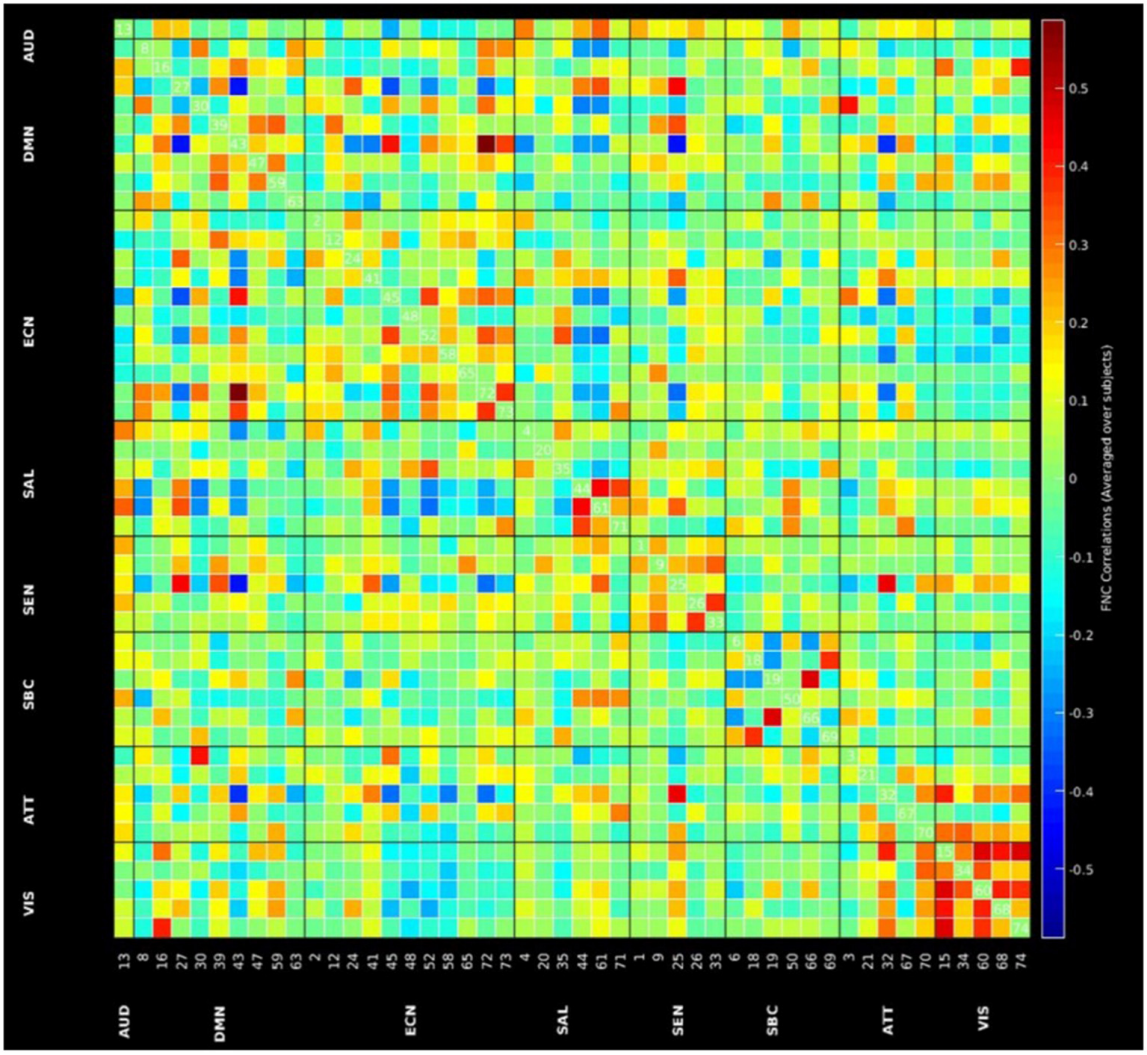
Inter-network functional network connectivity matrix of the 48 RSNs.

Figure 1 shows the spatial maps of the 48 selected RSNs. The 48 RSNs listed in Table 2 were grouped into eight domains: auditory (AUD), default mode network (DMN), executive control (ECN), salience (SAL), sensorimotor (SEN), subcortical (SBC), attentional (ATT), and visual (VIS) based on their peak coordinate, functional properties, the automatic labeling tool in GIFT, and confirmed by visual inspection (see Figure 2 for the estimated inter-network connectivity between domains and RSNs)^4^. Consistent with prior literature, the inter-network connectivity in Figure 2 suggests largely positive within domain inter-network connectivity within the DMN, ECN, SAL, ATT, and VIS networks (Espinoza et al., 2018; Du et al., 2020). Similar to analyses in clinical populations, Figure 2 also suggests cases of negative inter-network connectivity within SBC networks (Du et al., 2020).

**Table 2:** Resting-state networks (RSNs) domain names, IC numbers, and MNI peak coordinates.

**RSNs and domain names**	**IC number**	**MNI peak (*x, y, z)***
**Auditory (AUD)**		
Left superior temporal gyrus	13	(−58, −22, 10)
**Default mode network (DMN)**		
Anterior cingulate	8	(2, 14, −5)
Posterior cingulate	16	(14, −56, 5)
Right postcentral gyrus	27	(56, −32, 55)
Right inferior temporal gyrus	30	(54, −24, −20)
Precuneus	39	(0, −66, 60)
Precuneus	43	(0, −66, 35)
Posterior cingulate	47	(0, −60, 10)
Right angular gyrus	59	(38, −80, 30)
Right parahippocampal gyrus	63	(22, −28, −10)
**Executive control network (ECN)**		
Left middle frontal gyrus	2	(−38, 44, −5)
Right inferior parietal lobule	12	(48, −58, 55)
Right inferior frontal gyrus	24	(48, 14, 30)
Superior frontal gyrus	41	(2, 14, 70)
Superior frontal gyrus	45	(0, 44, 55)
Left superior frontal gyrus	48	(−12, −4, 70)
Right superior frontal gyrus	52	(24, 36, 30)
Right middle frontal gyrus	58	(24, 20, 50)
Left superior parietal lobule	65	(−38, −64, 55)
Medial frontal gyrus	72	(0, 52, −5)
Medial frontal gyrus	73	(0, 62, 5)
**Salience network (SAL)**		
Left superior temporal gyrus	4	(−42, 8, −15)
Left middle frontal gyrus	20	(−42, 52, 10)
Cingulate gyrus	35	(0, 10, 35)
Right inferior frontal gyrus	44	(44, 16, 5)
Medial frontal gyrus	61	(2, 0, 55)
Anterior cingulate	71	(0, 32, 35)
**Sensorimotor (SEN)**		
Right precentral gyrus	1	(54, −8, 30)
Left precentral gyrus	9	(−48, −36, 60)
Postcentral gyrus	25	(10, −60, 70)
Medial frontal gyrus	26	(0, −22, 70)
Right precentral gyrus	33	(42, −22, 65)
**Subcortical (SBC)**		
Basal ganglia	6	(0, −22, −5)
Left lentiform nucleus	18	(−18, −10, 0)
Left parahippocampal gyrus	19	(−18, −20, −20)
Left lentiform nucleus	50	(−24, 4, 0)
Right parahippocampal gyrus	66	(18, −4, −15)
Right lentiform nucleus	69	(18, −8, 0)
**Attentional (ATT)**		
Left superior temporal gyrus	3	(−36, 10, −30)
Left middle temporal gyrus	21	(−60, −30, 0)
Precuneus	32	(4, −82, 45)
Left supramarginal gyrus	67	(−60, −48, 40)
Right middle temporal gyrus	70	(56, −66, 5)
**Visual (VIS)**		
Cuneus	15	(2, −94, 25)
Right inferior occipital gyrus	34	(32, −94, −10)
Right fusiform gyrus	60	(38, −68, −20)
Left lingual gyrus	68	(−24, −66, −10)
Lingual gyrus	74	(0, −80, −5)

RSN network names were determined by peak MNI coordinates and gICA's component labeling function.

### Time-course power spectra

#### PCL-R factor 1 scores

**Figure 3:**
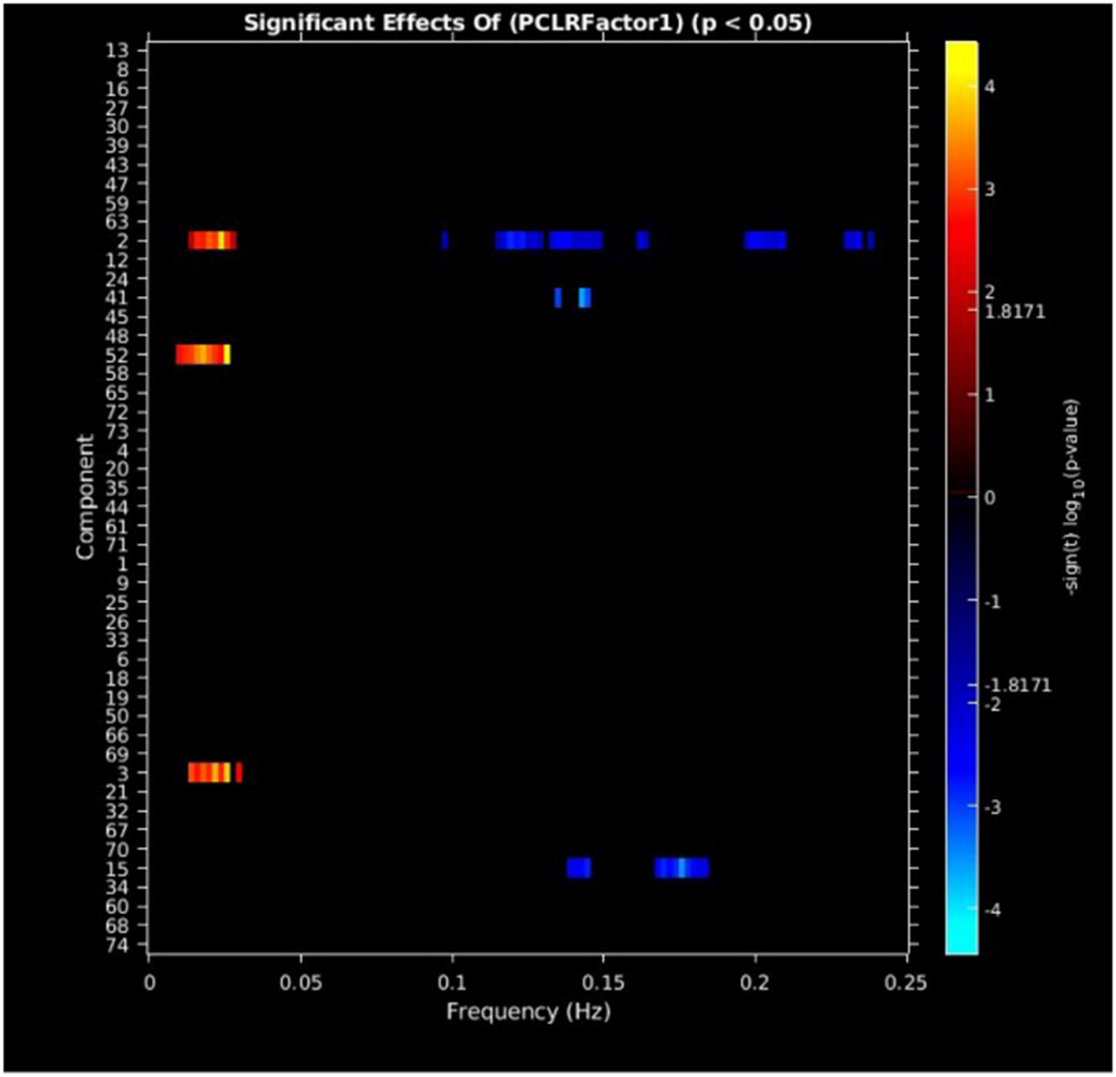
Univariate associations between PCL-R Factor 1 score and power spectra of significant components, predominantly occurring in the ECN. Panel depicts the significance and direction of PCL-R Factor 1 scores as a function of frequency for each significant component, displayed as–sign(t) log10(p), FDR corrected *p* < 0.05.

PCL-R Factor 1 scores were associated with increased AF at low-frequency bands (0–0.05 Hz) in the left middle frontal gyrus (Component 2, ECN), the left superior temporal gyrus (Component 3, ATT), and the right superior frontal gyrus (Component 52, ECN), and decreased AF at high-frequency spectra bands (0.09–0.25 Hz) in the cuneus (Component 15, VIS), left middle frontal gyrus (Component 2, ECN), and the superior frontal gyrus (Component 41, ECN) (see Figures 3, 5, Table 3).

**Table 3:** Effects of psychopathic traits on AFs, FDR corrected.

**Measure**	**RSN**	**IC, domain**	**Beta range**
**PCL-R factor 1**			
	Left middle frontal gyrus	2, ECN	−0.0433 to 0.0327
	Left superior temporal gyrus	3, ATT	0.0451 to 0.0543
	Right superior frontal gyrus	52, ECN	0.0468
	Cuneus	15, VIS	−0.0489 to −0.0459
	Superior frontal gyrus	41, ECN	−0.0495 to −0.0443
**PCL-R factor 2**			
	Posterior cingulate cortex	47, DMN	−0.0459 to −0.0403
	Left middle frontal gyrus	2, ECN	−0.0337 to −0.0317

Table shows all AF effects that survive FDR correction at p < 0.05 level and range of Beta Values per component.

#### PCL-R factor 2 scores

**Figure 4:**
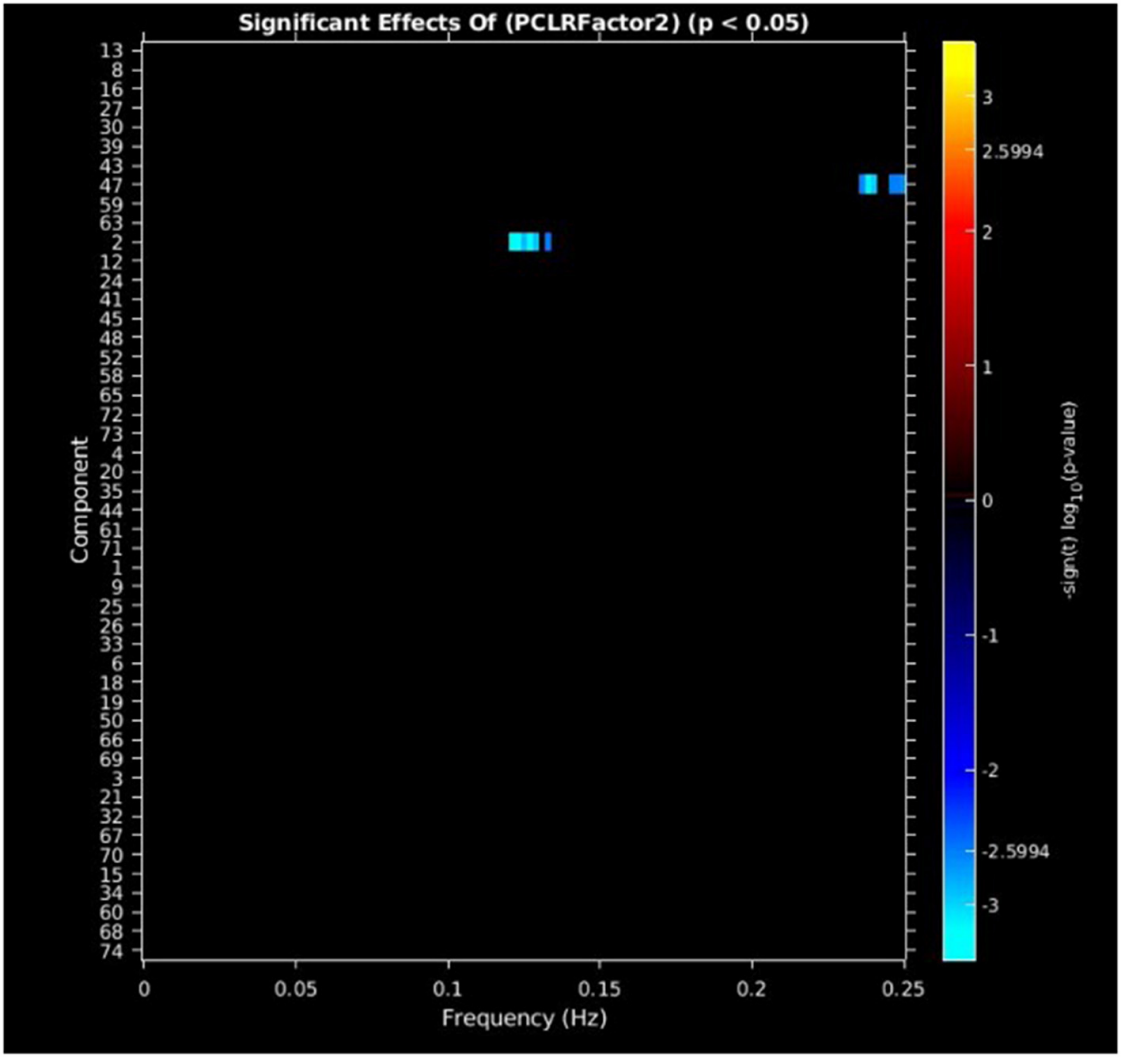
Univariate associations between PCL-R Factor 2 score and power spectra of significant components. Panel depicts the significance and direction of PCL-R Factor 2 scores as a function of frequency for the significant component, displayed as–sign(t) log10(p), FDR corrected *p* < 0.05.

**Figure 5:**
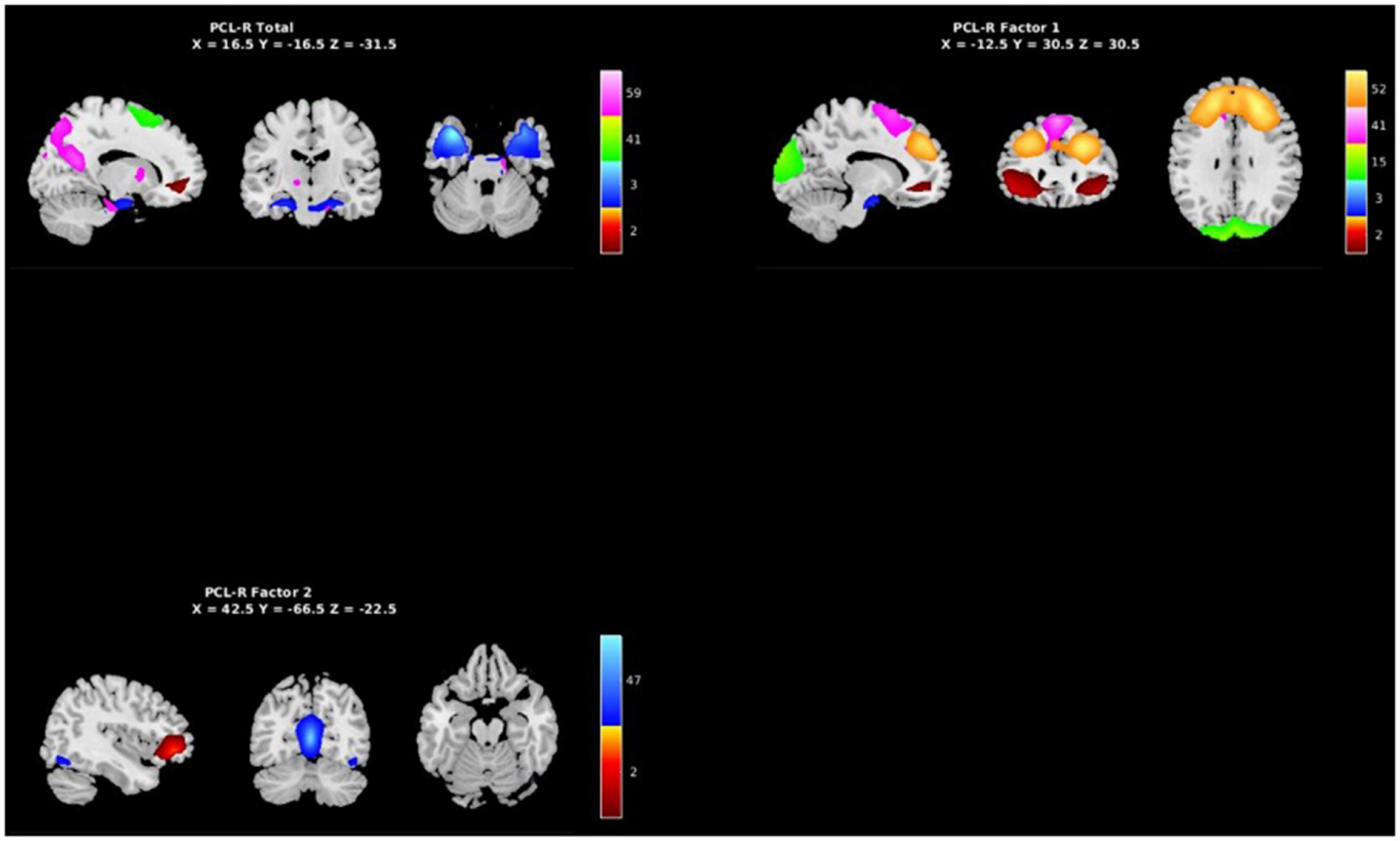
Spatial maps of the 7 independent components identified in Table 3 as exhibiting psychopathy related aberrant AFs, grouped by PCL-R Total effects (see Supplementary materials), PCL-R Factor 1 effects, and PCL-R Factor 2 effects.

PCL-R Factor 2 scores were associated with decreased AF at high-frequency bands (0.10–0.25 Hz) in the posterior cingulate cortex (Component 47, DMN) and left middle frontal gyrus (Component 2, ECN) (see Figures 4, 5, Table 3).

### Component spatial maps

#### PCL-R factor 1 scores

**Figure 6:**
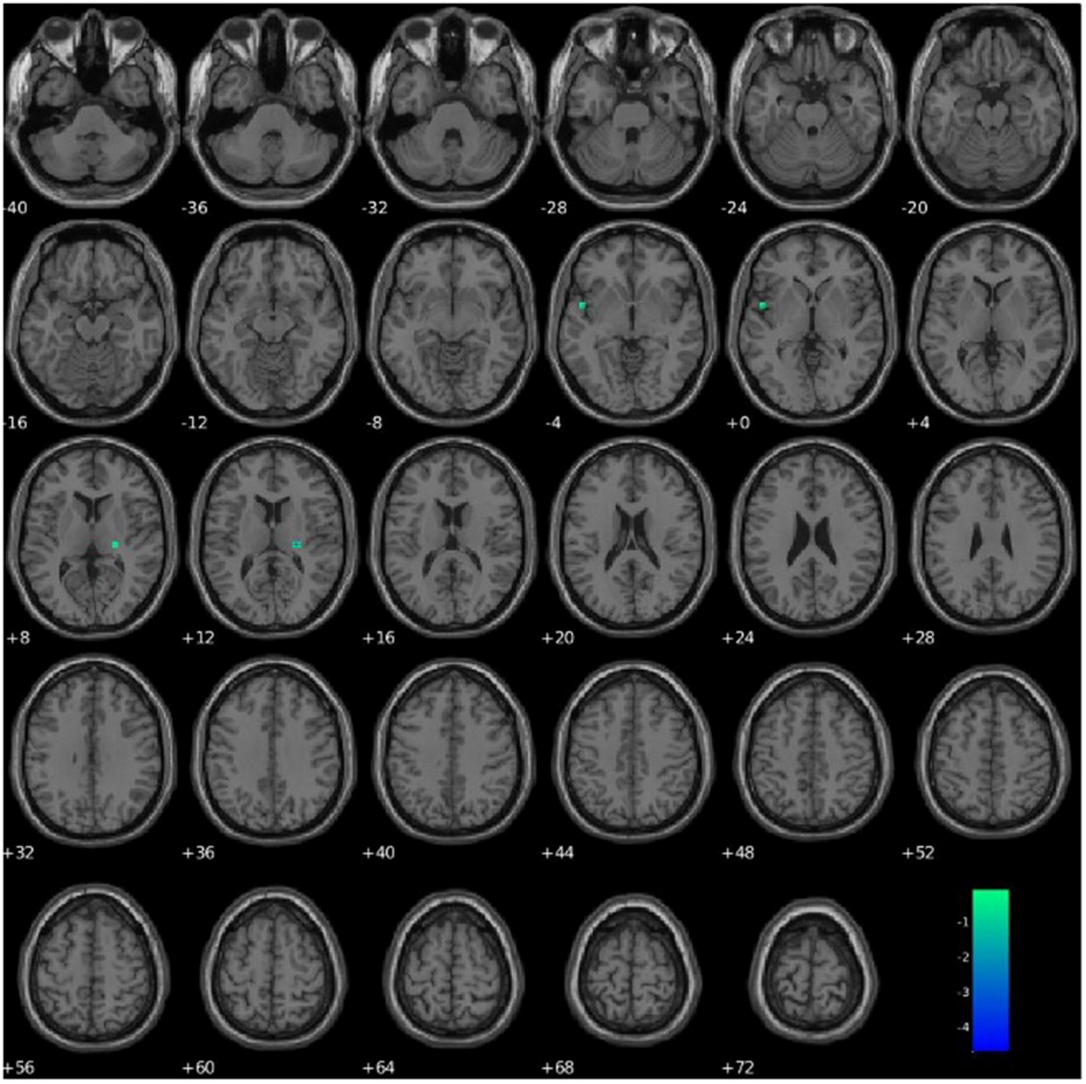
Association between PCL-R Factor 1 score and intra-network connectivity within a network primarily pertaining to the parahippocampal gyrus, FDR corrected *p* < 0.05.

PCL-R Factor 1 scores were associated with functional connectivity in a network primarily pertaining to the parahippocampal gyrus (Component 63, DMN), such that higher PCL-R Factor 1 scores were associated with decreased intra-network functional connectivity of the left insula and right thalamus within Component 63 (see Figure 6, Table 4).

**Table 4:** Effects of psychopathic traits on intra-network connectivity, FDR corrected.

**Measure**	**RSN**	**IC, domain**	**Average beta**
**PCL-R factor 1**			
	Right parahippocampal gyrus	63, DMN	−0.4143
**PCL-R factor 2**			
	Right fusiform gyrus	60, VIS	0.2403, −0.2654
	Left lingual gyrus	68, VIS	0.2688

Table shows all clusters that survive FDR correction at p < 0.05 level and average Beta effect size per component.

#### PCL-R factor 2 scores

**Figure 7:**
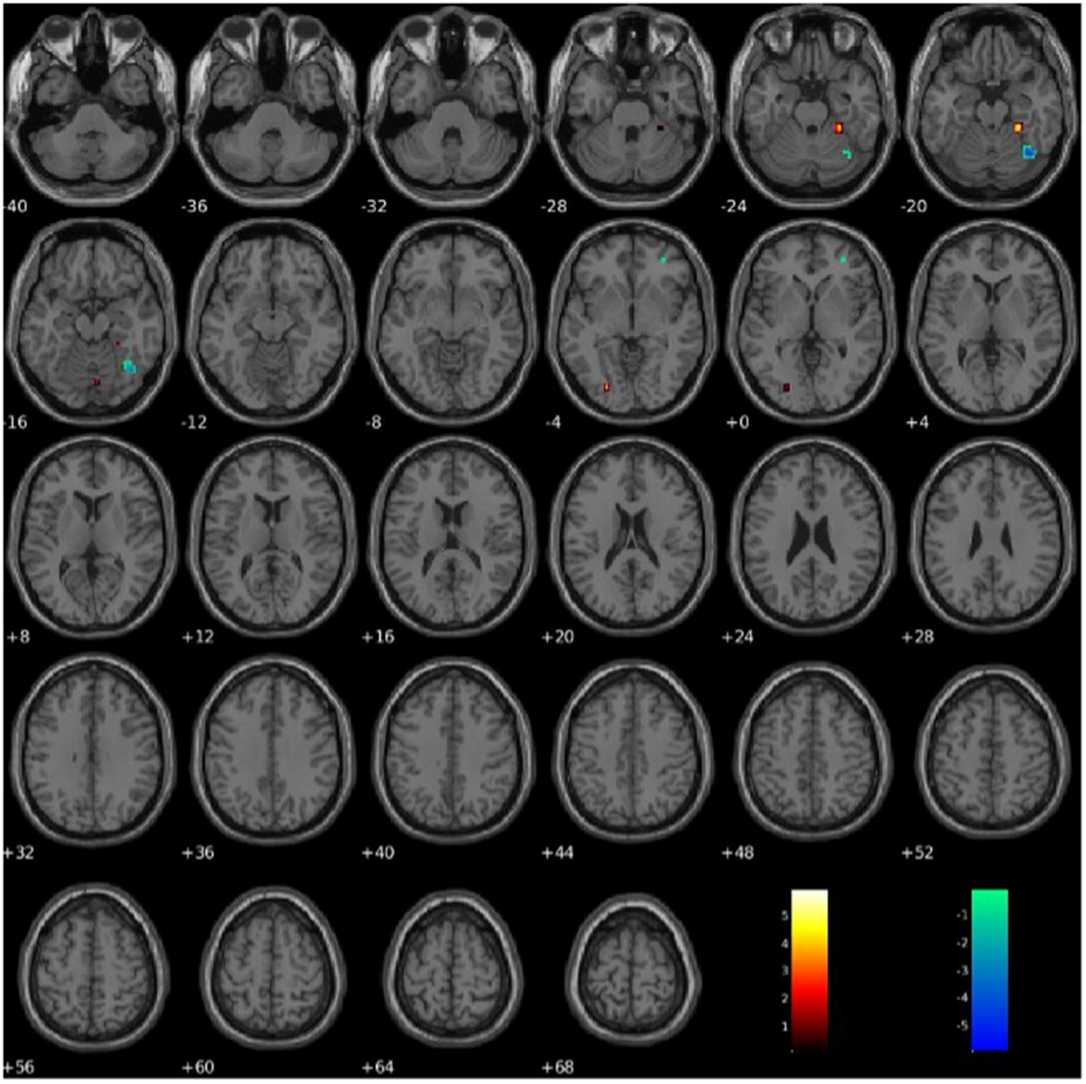
Association between PCL-R Factor 2 score and intra-network connectivity within the lingual gyrus and fusiform gyrus, FDR corrected *p* < 0.05.

PCL-R Factor 2 scores were associated with functional connectivity in the right fusiform gyrus (Component 60, VIS), such that higher PCL-R Factor 2 scores were associated with both increased and decreased intra-network functional connectivity (see Figure 7). PCL-R Factor 2 scores were also associated with functional connectivity in the left lingual gyrus (Component 68, VIS), such that higher PCL-R Factor 2 scores were associated with an increased intra-network functional connectivity (see Figure 7, Table 4).

### Functional network connectivity

There were no significant associations between PCL-R scores and sFNC that survived FDR correction while controlling for age, IQ, and FWD.

## Discussion

Here we report that psychopathic traits (assessed *via* the PCL-R) were associated with aberrant functional connectivity measures during a resting-state fMRI experimental paradigm in a sample of incarcerated women. Consistent with previous research performed in men and our hypotheses, PCL-R scores were associated with aberrant functional connectivity across multiple domains among incarcerated women.

PCL-R scores were associated with increased amplitude fluctuations (AF) in low-frequency bands and reduced AF in high-frequency bands across regions included within the paralimbic system, including the PCC and superior temporal gyrus, and additional regions, including the superior frontal gyrus, middle frontal gyrus, and cuneus. Compared to low-frequency AFs, high-frequency AFs are believed to contribute to higher-order cognitive processes (Baria et al., 2011; Craig et al., 2018). Reduced high-frequency AFs may relate to some of the previously observed deficits characteristic of women scoring high on psychopathy. For example, successful error-related processing depends on the collaboration of several higher-order brain regions, including the middle/superior frontal gyrus, PCC, and cuneus (Steele et al., 2014). Women scoring high on the PCL-R have been previously characterized by error-related processing deficits, including reduced amplitude of the error-related positivity event-related potential component (Maurer et al., 2016). Furthermore, women scoring high on psychopathy have been previously characterized by reduced reactivity to emotional facial expressions (Eisenbarth et al., 2013); regions implicated in the current study, including the middle frontal gyrus, have been previously associated with processing of emotional faces (Pessoa et al., 2002; Willis et al., 2010).

We also observed that women scoring high on psychopathy were characterized by aberrant intra-network connectivity within regions of the paralimbic system (e.g., parahippocampal cortex) and additional regions, including the fusiform gyrus and lingual gyrus. Women scoring high on psychopathy were characterized by reduced intra-network functional connectivity within Component 63 (parahippocampal gyrus), Component 60 (right fusiform gyrus), and Component 68 (left lingual gyrus), and increased intra-network functional connectivity in Component 60 (right fusiform gyrus). Reduced intra-network functional connectivity in the fusiform and parahippocampal gyrus is consistent with a previously published study with incarcerated women, observing that women scoring higher on the PCL-R were characterized by reduced hemodynamic activity in the fusiform and parahippocampal gyrus during the processing of moral violations (Harenski et al., 2014b). Our finding of PCL-R Factor 2 relating to increases in intra-network functional connectivity of the fusiform and lingual region is also consistent with previously published studies suggesting relationships between impulsivity and intra-visual network functional connectivity (Davis et al., 2013; Pu et al., 2017).

Published studies investigating resting-state AFs and youth psychopathic traits have reported different findings than those obtained in the current study. For example, Thijssen and Kiehl (2017) observed that youth scoring high on the Psychopathy Checklist: Youth Version (PCL:YV; Forth and Kosson, 2003) were largely characterized by *decreased* AFs in low-frequency bands and *increased* AFs at high-frequency bands. While differences between studies may have been observed, we believe that the results obtained in the current study may relate to specific deficits previously observed in women scoring high on psychopathy. For example, increased low frequency AFs are believed to relate to improvements within neural efficiency (Biswal et al., 1995). Compared to men scoring high on psychopathy, women scoring high on psychopathy are not characterized by the response perseveration deficits (Vitale and Newman, 2001a). Important to note, response perseveration is associated with dysfunction with several regions implicated in the current study, including the middle frontal gyrus (Yang et al., 2011), superior temporal gyrus (Ersche et al., 2011), and superior frontal gyrus (De Ruiter et al., 2009; Camchong et al., 2011). Therefore, women scoring high on psychopathy may be characterized by increased neural efficiency (observed via higher low-frequency AFs) in several paralimbic brain regions, contributing to improved performance on response perseveration tasks (Vitale et al., 2011). By exhibiting reduced AFs in low-frequency bands, this may relate to youth scoring high on psychopathy exhibiting response preservation deficits (Vitale et al., 2005).

Importantly, and more broadly, our investigation into the relationships between psychopathic traits in women and multiple measurements of rsFNC (inter-network connectivity, intra-network connectivity, and AFs) underscore three points. First, neurobiological aberrances related to psychopathic traits in women may best be accounted for on a local (i.e., intra-network connectivity & AFs) neurobiological level rather than a global level (i.e., inter-network connectivity). Second, while variability in psychopathic traits may be accounted for by the relationships between voxel and RSN time courses (e.g., Thijssen and Kiehl, 2017; Espinoza et al., 2018), additional variability can also be explained by rates and amplitudes of individual RSN activational profiles themselves (i.e., AFs; see Thijssen and Kiehl, 2017). And finally, by exploring both local (i.e., intra-network connectivity and AFs) and global (i.e., inter-network connectivity) rsFNC measures in their relationships to antisocial traits, future research stands to further explore and potentially differentiate how antisocial phenotypes are represented neurobiologically across various demographic groups.

### Study limitations

A number of limitations must be considered for the present study. Though all regions identified as being associated with psychopathic traits in the present study were also identified in Espinoza et al. (2018), here we did not find any significant psychopathy related inter-network FNC results. One potential explanation for this result is the size of the sample analyzed. While the present sample is considered large by neuroimaging standards (*n* = 297), Espinoza et al. (2018) conducted inter-network connectivity analysis in a sample more than three times as large (*n* = 985). Additionally, because all FNC measures tested in the present analysis were static, there are a number of assumptions being made regarding the relationships between the consistency of network activity across the 5-min resting-state scan. Similarly, because the scans were resting-state rather than task-based, extrapolations of RSN aberrances to specific functional domains are hard to attribute. Finally, while our experimental design utilized 5-min resting-state scans, there is evidence suggesting that longer scans are needed to ensure higher RSN stabilities (e.g., Birn et al., 2013; though see Allen et al., 2011a; Espinoza et al., 2018, 2019; Duda et al., 2022). Thus, more work is needed to probe the relationships between various inter- and intra-network static and dynamic connectivity measures as they relate to psychopathic traits and perhaps longer resting-state and task-based measures in large samples of both men and women.

## Conclusion

This study contributes to the current literature by examining whole brain inter- and intra-network connectivity and AFs across RSNs and their relationship to psychopathic traits in women. We showed that psychopathy is associated with increased low-frequency, decreased high-frequency AFs, and both increased and decreased intra-network connectivity across four brain domains (DMN, ECN, VIS, and ATT). Similar to previous analyses in incarcerated men, our results suggest that psychopathic traits among incarcerated women are associated with aberrant intra-network AFs and connectivity across multiple networks associated with executive control, decision making, salience detection, and motor control. Our results showcase aberrant intra-network FNC rather than aberrant inter-network FNC underlying psychopathic traits in women, suggesting the potential for sex-specific neurobiological psychopathy related phenotypes. To our knowledge, this represents the largest study to date on the association of psychopathic traits and intrinsic RSN aberrances in incarcerated women.

## Data availability statement

The datasets presented in this article are not readily available because of ethical and privacy restrictions. Requests to access the datasets should be directed to KK, kkiehl@mrn.org.

## Ethics statement

The studies involving human participants were reviewed and approved by University of New Mexico IRB and the Independent Review (E&I) Services for the Mind Research Network. The patients/participants provided their written informed consent to participate in this study.

## Author contributions

CA performed the statistical analysis and wrote the first draft of the manuscript. JM wrote sections of the manuscript. All authors contributed to the conception, design of the study, manuscript revision, read, and approved the submitted version.

## Funding

This material is based upon work supported by the National Institute on Drug Abuse (NIDA; R01 DA020870 and R01 DA026964) and National Institute of Mental Health (NIMH; R01 MH085010).

## Conflict of interest

Authors CA, JM, BE, AG, CH, KH, and KK were employed by The Mind Research Network.

The remaining author declares that the research was conducted in the absence of any commercial or financial relationships that could be construed as a potential conflict of interest.

## Publisher's note

All claims expressed in this article are solely those of the authors and do not necessarily represent those of their affiliated organizations, or those of the publisher, the editors and the reviewers. Any product that may be evaluated in this article, or claim that may be made by its manufacturer, is not guaranteed or endorsed by the publisher.

## Author disclaimer

The contents of this manuscript are solely the responsibility of the authors and do not necessarily represent the views of the National Institute on Drug Abuse nor the National Institute of Mental Health.

## Footnotes

^1^One participant in the sample was administered the SCID-5-RV, whereas the rest were administered the SCID-I/P.

^2^As estimated by the vocabulary and matrix reasoning subscales of the Wechsler Adult Intelligence Scale; see Ryan and Ward, 1999.

^3^Note: The inclusion or exclusion of FWD in analyses did not alter significant findings for any present analyses.

^4^While the automatic labels and correlations to networks provided by the GIFT toolbox (http://trendscenter.org/software/gift) (Calhoun et al., 2001) were helpful in the initial network classification and description, these network labels differ from those commonly utilized in relevant literature (i.e., Espinoza et al., 2018). Thus, a combination of the correlational outputs/labeling, the peak coordinates, and functional properties commonly ascribed to the regions were used to name regions and assign those regions to relevant domains. The assignment of regions to a specific domain was performed independently by three authors and the final domain classification was based upon unanimous agreements.
