# Supplementary material for: Aberrant resting-state functional connectivity in incarcerated women with elevated psychopathic traits
Source: Front Neuroimaging. 2022 Oct 4;1:971201. doi: 10.3389/fnimg.2022.971201 (PMC10406317; doi:10.3389/fnimg.2022.971201)
Supplement: Supplementary file 1 [file Data_Sheet_1.docx]

Supplementary Material

# Supplementary Analyses: PCL-R Total

## Time-course Power Spectra

### PCL-R Total Scores

PCL-R total scores were associated with increased AF in low-frequency bands (0Hz - 0.05Hz) in the left middle frontal gyrus (Component 2, ECN), superior frontal gyrus (Component 41, ECN), and the left superior temporal gyrus (Component 3, ATT), and decreased AF at high-frequency bands (0.10Hz - 0.21Hz) in the left middle frontal gyrus (Component 2, ECN), superior frontal gyrus (Component 41, ECN), and right angular gyrus (Component 59, DMN) (see Figures S1 and 5, Table S1).

## Component Spatial Maps

### PCL-R Total Scores

PCL-R total scores were associated with functional connectivity in the right fusiform gyrus (Component 60, VIS), such that higher PCL-R total scores were associated with increased intra-network functional connectivity within Component 60 (see Figure S2, Table S2).

## Supplementary Tables

## Table S1. Effects of psychopathic traits total on AFs, FDR corrected.

| **Measure** | **RSN** | **IC, domain** | **Beta Range** |
| --- | --- | --- | --- |
| **PCL-R Total** |  |  |  |
|  | Left middle frontal gyrus | 2, ECN | -0.0224 - 0.0381 |
|  | Superior frontal gyrus | 41, ECN | -0.0245 - 0.0163 |
|  | Left superior temporal gyrus | 3, ATT | 0.0237 |
|  | Right angular gyrus | 59, DMN | -0.0220 |

## Table S2. Effects of psychopathic traits total on intra-network connectivity, FDR corrected.

| **Measure** | **RSN** | **IC, domain** | **Average Beta** |
| --- | --- | --- | --- |
| **PCL-R Total Score** |  |  |  |
|  | Right fusiform gyrus | 60, VIS | 0.1448 |

**
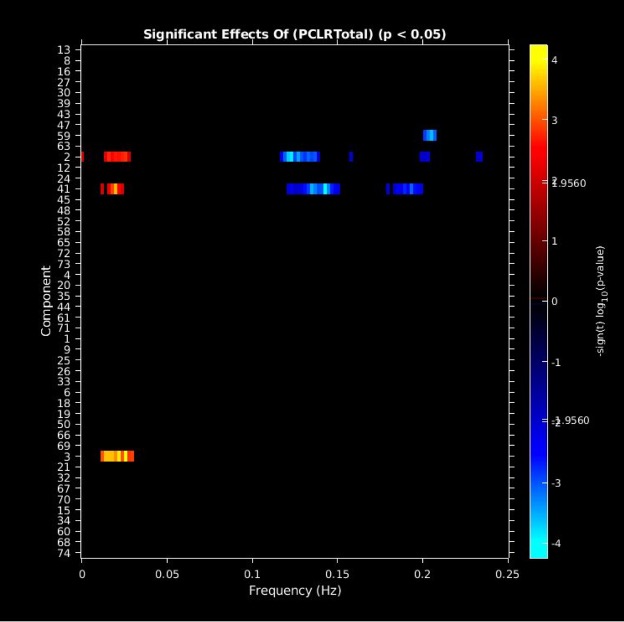
**

**Supplementary Figure 1** Univariate associations between PCL-R Total score and power spectra of significant components, predominantly occurring in the ECN. Panel depicts the significance and direction of PCL-R Total scores as a function of frequency for each significant component, displayed as - sign(t)log10(p), FDR corrected *p* < 0.05.


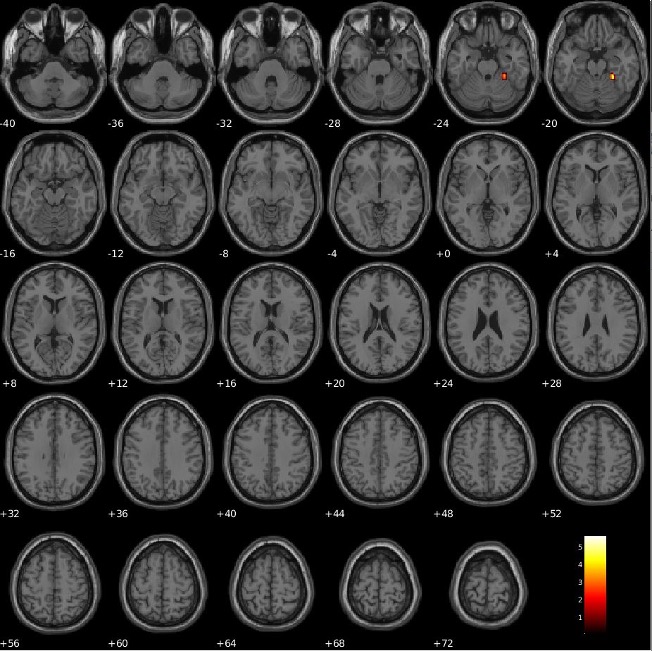


**Supplementary Figure 2** Association between PCL-R Total score and intra-network connectivity within the fusiform gyrus, FDR corrected *p* < 0.05.
